# Novel compound heterozygous mutations of *CLDN16* in a patient with familial hypomagnesemia with hypercalciuria and nephrocalcinosis

**DOI:** 10.1002/mgg3.1475

**Published:** 2020-09-01

**Authors:** Alejandro García‐Castaño, Ana Perdomo‐Ramirez, Mònica Vall‐Palomar, Elena Ramos‐Trujillo, Leire Madariaga, Gema Ariceta, Felix Claverie‐Martin

**Affiliations:** ^1^ Biocruces Bizkaia Research Institute Barakaldo Bizkaia Spain; ^2^ Unidad de Investigación Hospital Universitario Nuestra Señora de Candelaria Santa Cruz de Tenerife Spain; ^3^ Fisiopatologia Renal Centro de Investigaciones en Bioquímica y Biología Molecular (CIBBIM) Vall d’Hebron Institut de Recerca (VHIR Barcelona Spain; ^4^ Pediatric Nephrology Department Cruces University Hospital UPV/EHU Barakaldo Spain; ^5^ Servicio de Nefrología Pediátrica Hospital Universitari Vall d'Hebron Barcelona Spain; ^6^ Departamento de Pediatría Universitat Autònoma de Barcelona Bellaterra (Barcelona) Spain

**Keywords:** claudin‐16, *CLDN16*, deletion, hypomagnesemia, novel mutations, QMPSF

## Abstract

**Background:**

Familial hypomagnesemia with hypercalciuria and nephrocalcinosis (FHHNC) is an autosomal recessive tubulopathy characterized by excessive urinary wasting of magnesium and calcium, bilateral nephrocalcinosis, and progressive chronic renal failure in childhood or adolescence. FHHNC is caused by mutations in *CLDN16* and *CLDN19*, which encode the tight‐junction proteins claudin‐16 and claudin‐19, respectively. Most of these mutations are missense mutations and large deletions are rare.

**Methods:**

We examined the clinical and biochemical features of a Spanish boy with early onset of FHHNC symptoms. Exons and flanking intronic segments of *CLDN16* and *CLDN19* were analyzed by direct sequencing. We developed a new assay based on Quantitative Multiplex PCR of Short Fluorescent Fragments (QMPSF) to investigate large *CLDN16* deletions.

**Results:**

Genetic analysis revealed two novel compound heterozygous mutations of *CLDN16*, comprising a missense mutation, c.277G>A; p.(Ala93Thr), in one allele, and a gross deletion that lacked exons 4 and 5,c.(840+25_?)del, in the other allele. The patient inherited these variants from his mother and father, respectively.

**Conclusions:**

Using direct sequencing and our QMPSF assay, we identified the genetic cause of FHHNC in our patient. This QMPSF assay should facilitate the genetic diagnosis of FHHNC. Our study provided additional data on the genotypic spectrum of the *CLDN16* gene.

## Graphical abstract

We report the clinical and genetic findings of a child diagnosed with FHHNC, a rare autosomal recessive disease characterized by excessive renal magnesium and calcium wasting, bilateral nephrocalcinosis, and progressive chronic renal failure. Two novel compound heterozygous mutations of *CLDN16*, c.277G>A, p.(A93T) and a gross deletion involving exons 4 and 5, inherited from the patient's parents, were identified. We developed a new assay based on Quantitative Multiplex PCR of Short Fluorescent Fragments, which should facilitate the genetic diagnosis of FHHNC.

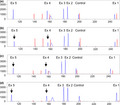

## 1 INTRODUCTION

Loss‐of‐function mutations in two genes involved in renal paracellular reabsorption of calcium and magnesium, *CLDN16* (OMIM *603959) and *CLDN19* (OMIM *610036), cause two types of a rare autosomal recessive disease known as Familial Hypomagnesemia with Hypercalciuria and Nephrocalcinosis, respectively (FHHNC; type 1, OMIM #248250 and type 2, #248190) (Konrad et al., 2006; Simon et al., 1999). This tubulopathy is characterized by excessive urinary losses of renal magnesium and calcium, bilateral nephrocalcinosis, and progressive chronic renal failure early in life (Claverie‐Martin, 2015; Praga et al., 1995). Patients usually present with recurrent urinary tract infections, polyuria, polydipsia, and nephrolithiasis. The symptoms usually appear in early childhood or adolescence. Common biochemical characteristics include low levels of magnesium in serum, high urine levels of calcium and magnesium, high serum levels of parathyroid hormone, and reduced glomerular filtration rate (Godron et al., 2012; Konrad et al., 2008; Weber et al., 2001; Claverie‐Martin et al., 2013). Defects in enamel formation have been reported in some FHHNC patients (Bardet et al., 2016; Yamaguti et al., 2017). In addition, patients with mutations in *CLDN19* display severe ocular abnormalities such as myopia, macular colobamata, and nystagmus (Konrad et al., 2006; Claverie‐Martin et al., 2013; Godron et al., 2012).

*CLDN16* and *CLDN19* encode tight junction proteins claudin‐16 and claudin‐19, respectively, which are members of a family of membrane proteins that contain four transmembrane domains, two extracellular loops, a cytoplasmic loop, and cytoplasmic amino‐ and carboxy ends (Meoli & Günzel, 2020; Suzuki et al., 2014). Both claudins interact with each other on adjacent epithelial cells of the thick ascending loop of Henle to form a cation paracellular selective barrier that regulates the paracellular reabsorption of magnesium and calcium in the kidney (Hou et al., 2008). Claudin‐16 and claudin‐19 are also expressed in the tight junction of ameloblasts, which might explain their association with defective enamel formation in FHHNC patients (Bardet et al., 2016; Yamaguti et al., 2017). Moreover, severe ocular involvement in FHHNC patients with mutation in claudin‐19 might be attributed to the expression of this claudin in fetal retinal pigment epithelium (Peng, Rao, Adelman, & Rizzolo, 2011; Wang et al., 2019).

The clinical diagnosis of FHHNC needs to be confirmed by the detection of *CLDN16* or *CLDN19* pathogenic variants in both alleles. Most *CLDN16* and *CLDN19* variants identified in FHHNC patients are missense mutations found in homozygous or compound heterozygous state (Claverie‐Martin et al., 2015; Prot‐Bertoye & Houillier, 2020). These mutations are mainly located in the two extracellular loops but a few affect the transmembrane domains and the cytoplasmic regions. Only one large deletion has been identified in the *CLDN16* gene of a patient with FHHNC (Yamaguti et al., 2015).

In this study, we aimed to describe the clinical and genetic features of a patient with early onset of the first FHHNC symptoms. Since direct DNA sequencing of coding exons and flanking intronic sequences only detected a missense heterozygous mutation of *CLDN16*, we designed a Quantitative Multiplex PCR of Short Fluorescent Fragments (QMPSF) assay to identify a novel large deletion in the other allele.

## 2 MATERIALS AND METHODS

### 2.1 Ethical compliance

The Ethics Committee of Hospital Universitario Nuestra Señora de Candelaria (Santa Cruz de Tenerife, Spain) approved the protocols of this study, which was conducted according to the Declaration of Helsinki. Written informed consent was obtained from the patient's parents.

### 2.2 Genomic DNA purification and direct sequence analysis

After obtaining written informed consent, peripheral blood samples of patients and relatives were collected for genetic analysis. Genomic DNA was extracted using the GenElute Blood Genomic DNA kit (Sigma‐Aldrich, St. Louis, MO, USA) following the manufacturer's instructions. The coding exons and flanking intronic sequences of *CLDN16* and *CLDN19* were amplified by polymerase chain reaction (PCR) using primers and conditions previously described (Claverie‐Martin et al., 2013; Perdomo‐Ramirez et al., 2019; Simon et al., 1999). PCR products were purified with the QIAquick PCR purification kit (Qiagen, Hilden, Germany) and sequenced with the BigDye Terminator v3.1 Cycle Sequencing Kit (Applied Biosystems, Foster City, CA, USA). Sequence reactions were purified with Performa^®^DTR Gel Filtration Cartridges (EdgeBio BioSystems, Gaithersburg, Maryland, USA), and analyzed on a 3500 Series Genetic Analyzer (Applied Biosystems, Foster City, CA, USA). Mutations were identified by comparison to the respective reference sequences (GenBank accession numbers NG_008993.1 and NG_008149.1, for CLD*N19* and *CLDN16*, respectively), and confirmed by sequencing additional independent amplification products. We inspected several databases, including ClinVar (https://www.ncbi.nlm.nih.gov/clinvar/), Human Gene Mutation database (HGMD, http://www.hgmd.cf.ac.uk/ac/index.php), 1000 Genomes Project (http://www.1000genomes.org/), and gnomAD database (https://gnomad.broadinstitute.org/), to verify that the variant detected in our patient was not a common polymorphism and to confirm that it was novel. There are two potential start codons (methionine 1 and methionine 71) in the *CLDN16* gene that would produce a short (235 amino acids) or a long (305 amino acids) claudin‐16 isoform (Hou, Paul, & Goodenough, 2005; Weber et al., 2001). It is currently unknown whether both isoforms are functional. For all *CLDN16* mutations described in the literature, nucleotide numbering starts with the A of the first ATG translation initiation site as nucleotide 1 (see, table 4 of Prot‐Bertoye & Houillier, 2020, and HGMD). We used this same numbering for the two mutations described here (Reference NCBI sequence used for numbering: NM_006580.3). We followed the recommendations of the Human Genome Variation Society (http://varnomen.hgvs.org/) for the description of sequence variants.

### 2.3 Quantitative Multiplex PCR of Short Fluorescent Fragments analysis

In order to detect the potential gross deletions or duplications in the *CLDN16* region, we used a QMPSF assay. Primer pairs for amplification of short exonic fragments corresponding to exons 1–5 of the *CLDN16* gene and a sequence control of the *HNF1B* gene were designed (Table 1). Simultaneous PCR‐amplification was performed in a 20µL reaction mixture containing dNTPs (10 mM), MgCl2, 10% DMSO, KAPA Taq DNA Polymerase (Kapabiosystems, Boston, Massachusetts), dye‐labeled primers (0.3 µM of each primer), and 40 ng of genomic DNA. The amplification conditions consisted of an initial step of denaturation at 94°C for 5 min, followed by 24 cycles of denaturation at 94°C for 20 s, annealing at 60°C for 20 s, and extension at 72°C for 20 s. A final extension was performed at 72°C for 10 min. Then, the samples were denatured and loaded onto an ABI3130xl Genetic Analyzer (Life Technologies). We used the GeneMapper^®^ Software v. 4.0 (Applied Biosystems, California) for data analysis. Normalization was performed by dividing the peak height of each amplification product by the peak height of the control‐amplified product. Finally, the results obtained from the test samples were compared with the results from a healthy individual and positive control samples.

**TABLE 1:** Primer used for QMPSF analysis of the human *CLDN16* gene

Exon^a^	Forward primer (5′–3′)	Reverse primer (5′–3′)	Amplicon size (bp)
1	GACCACCACTAGCCCACAGT	56‐FAM‐TGGTACCTGGCAATGTGAAA	243
2	AATGCTTTTGATGGGATTCG	56‐FAM‐CCATTACAAACTGGACCGAAC	185
3	TGGTAACTCGAGCGTTGATG	56‐FAM‐GTTGCTAGTCCAGCCAGACC	181
4	CCAGGAATCATTGGCTCTGT	56‐FAM‐GAACAGCTCCAGCCAAAAAG	160
5	TTGGACCTGAGAGAAACTATCCTT	56‐FAM‐AGCATACATTTTGGCCGTCT	113
Control^b^	56‐FAM‐TGTCTAGTGAGGACCCTTGG	GGATCTCTCGTTGCTTTCTG	199

a Numbering is according to DNA sequence (Ensembl: ENST00000264734.2).

b *HNF1B* gene.

### 2.4 Bioinformatics analysis

The potential detrimental effect of the amino acid substitution on the structure and function of claudin‐16 was evaluated using the following bioinformatics tools, which are based on different principles: PolyPhen‐2 (http://genetics.bwh.harvard.edu/pph2/) (Adzhubei et al., 2010), Panther v.15.0 (http://www.pantherdb.org/tools/csnpScoreForm.jsp) (Tang & Thomas, 2016), MutPred2 (http://mutpred.mutdb.org/) (Pejaver, Mooney, & Radivojac, 2017), MutationTaster2 (http://www.mutationtaster.org/) (Schwarz, Cooper, Schuelke, & Seelow, 2014), and Fathmm‐XF (http://fathmm.biocompute.org.uk/fathmm‐xf/) (Rogers et al., 2018). The protein sequence of human claudin‐16 was obtained from the Uniprot database (https://www.uniprot.org/, entry identifier Q9Y5I7). Protein stability changes resulting from the missense variant were estimated using web‐based programs MUpro (http://mupro.proteomics.ics.uci.edu/) (Cheng, Randall, & Baldi, 2006) and I‐Mutant 3.0 (http://gpcr2.biocomp.unibo.it/cgi/predictors/I‐Mutant3.0/I‐Mutant3.0.cgi) (Capriotti, Fariselli, & Casadio, 2005). These tools provide the predicted free energy change value (DDG) and the sign of the prediction. The DDG value is calculated from the unfolding Gibbs free energy value of the mutant protein minus the unfolding Gibbs free energy value of the wild type (kcal/mol). A DDG value below 0 means that the stability of the protein has decreased, whereas a DDG superior to 0 means it has increased.

### 2.5 Protein modeling

There is no experimentally determined 3D structure available for claudin‐16 protein in the Protein Data Bank (PDB). Therefore, wild‐type and mutant claudin‐16 were modeled using the SWISS‐MODEL server (Waterhouse et al., 2018) (https://swissmodel.expasy.org/). Protein sequences in FASTA format were uploaded and modeled with the crystal structure of human claudin‐4 in complex with the C‐terminal fragment of *Clostridium perfringens* enterotoxin (PDBe: 5b2g.2) (Shinoda et al., 2016).

## 3 RESULTS

### 3.1 Clinical description

An 11 month old previously healthy boy consulted for fever and respiratory symptoms, and leukocyturia was identified incidentally. Urinary tract infection was suspected but the finding by renal ultrasound of severe and bilateral nephrocalcinosis led to additional diagnostic work‐up. His physical exam, height, and weight were normal by age (percentile >99), as well as his blood pressure. Previously unnoticed polyuria and hyposthenuria were also identified. Biochemistry serum tests revealed increased creatinine (0.61 mg/dl) [estimated Glomerular Filtration Rate (eGFR): 56 ml/min/1.73 m^2^)], without associated electrolytes anomalies (potassium 5.3 mEq/L, chloride 106 mEq/L, total calcium 10.2 mg/dl, and phosphorus 4.9 mg/dl) except mild hypomagnesemia (magnesium 1.54 mg/dl) and elevated uric acid (7.4 mg/dl), and remarkably significant high intact parathyroid hormone (175 pg/ml), for the degree of renal function impairment, with adequate 25‐hydroxy‐vitamin D levels (25 ng/dl). Acid‐base balance was preserved. Urine exam demonstrated increased urinary calcium and magnesium excretion: urinary calcium/creatinine (U_Ca/Cr_) 0.39 mg/mg, urinary magnesium/creatinine (U_Mg/Cr_) 0.21 mg/mg (at the high‐normal level but inappropriately increased for the level of serum magnesium), and elevated fractional excretion of magnesium (FE_Mg_, 8.2%). At the age of 2 years, under the clinical suspect of FHHNC, genetic analysis was performed (see, below). The patient's follow‐up 7 years later was consistent with FHHNC with moderate chronic kidney disease (serum creatinine 1.16 mg/dl (eGFR: 40 ml/min/1.73 m^2^), persistent hypomagnesemia despite oral supplements (magnesium 1.3 mg/dL) due to maintained hypermagnesiuria (U_Mg/Cr_ 0.27 mg/mg, FE_Mg_ 24%), associated with significant hypercalciuria (U_Ca/Cr_ 0.42 mg/mg; VCa 5.4 mg/Kg/day). His PTH levels were normalized under medical treatment. Dentistry careful evaluation did not demonstrate any enamel defects. Furthermore, the patient had no ocular abnormalities. His parents are not consanguineous, and there is no family history of nephropathy except a maternal great grandmother with a single kidney and nephrolithiasis.

### 3.2 Detection of a novel *CLDN16*missense mutation by direct DNA sequencing

**FIGURE 1:**
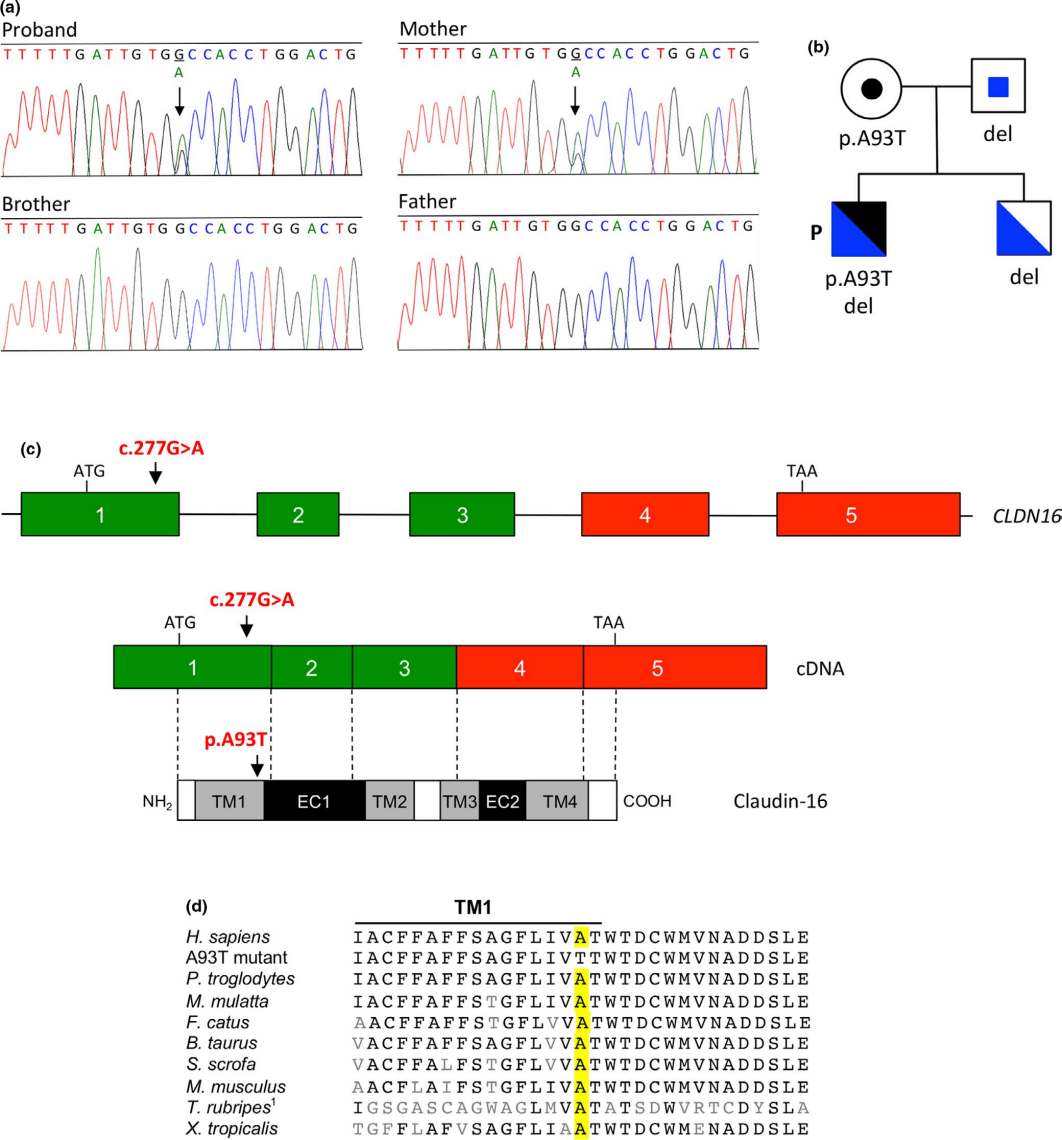
Identification of novel *CLDN16* heterozygous missense mutation c.277G>A; p.(Ala93Thr) in patient with FHHNC. (a) Electropherograms showing the heterozygous substitution in exon 1 of the patient and his mother. Arrows indicate the position affected by the mutation in the patient and his mother. The brother and father revealed the normal sequence. (b) Pedigree of the family showing segregation of both *CLDN16* variants (missense mutation and deletion in blue and black, respectively). Circle, female individual; squares, male individual; P, proband; del, c.(840+25_?)del detected in the other allele. The patient inherited the missense mutation and the deletion from his mother and father, respectively. (c) Schematic representation of the *CLDN16* gene, cDNA, and claudin‐16 protein. Arrows indicate the position affected by missense mutation c.277G>A; p.(Ala93Thr). Colored boxes represent the five coding exons and black lines indicate intron sequences; exons missing in the deletion mutant are in red. Exons and introns sizes are not at scale. The positions of the ATG start codon and the TAA stop codon are also shown. Exons are associated by dotted lines to the schematic representation of the claudin‐16 protein where transmembrane domains (TM1–TM4), extracellular segments (EC1 and EC2) and cytoplasmic regions (white boxes) are indicated. (d) Multiple sequence alignment of claudin‐16 or related proteins from different species showing evolutionary conservation of alanine 93. ^1^ *T*. *rubripes* does not contain the ortholog for mammalian claudin‐16. The sequence shown is that of claudin‐11B

DNA sequence analysis showed that the *CLDN19* coding exons and their flanking intronic regions had the normal sequence. Analysis of the *CLDN16* gene revealed a heterozygous variant c.277G>A, in exon 1 (Figure 1a). This variant was also detected in heterozygosis in the patient's mother, who was unaffected. Sequence analysis of the patient's father and brother, both healthy, showed the *CLDN16* normal sequence (Figure 1a). The pedigree of the family is shown in Figure 1b.The G to A substitution leads to the replacement of alanine for threonine in residue 93 of the claudin‐16 protein, p.(Ala93Thr). Alanine 93 is located next to the last amino acid of the first transmembrane domain (Figure 1c), and is highly conserved throughout the evolution (Figure 1d). We searched several databases to verify that the variant detected in our patient, c.277G>A; p.(Ala93Thr), was not a common SNP and to confirm that it was a novel mutation not previously reported. This variant was not found in ClinVar, HGMDor 1000 Genomes Project database. In the gnomAD database, it was found in only one allele from a genome sample from African population with a frequency of 0.00003 (variant ID: 3‐190106185‐G‐A). *In silico* prediction tools PolyPhen‐2, Panther, MutPred2, MutationTaster, and Fathmm‐XF indicated that this rare variant was probably pathogenic (Table 2). Furthermore, this variant caused a decrease in claudin‐16 protein stability according to programs that predict the protein stability of mutated proteins including I‐Mutant 3.0 (DDG in kcal/mol: −1.51).We submitted this new missense *CLDN16* mutation to ClinVar and it was included with accession number VCV000930213.1.

**TABLE 2:** Pathogenicity prediction for claudin‐16 mutation p.(Ala93Thr) using bioinformatics tools

Tool	PolyPhen‐2^a^	Panther^b^	MutPred2^c^	MutationTaster^d^	Fathmm‐XF^e^
Score	0.85	362	0.747	58	0.615
Significance	Possibly damaging	Possibly damaging	Probably pathogenic	Disease causing	Pathogenic

a The PolyPhen‐2 score varies from 0 to 1 Variants with scores in the range 0.85 to 1.0 are more confidently predicted to be damaging.

b Panther measures the length of time (in millions of years, my) an amino acid position in the protein has been preserved. The longer a position has been preserved, the more likely that amino acid change will have a deleterious effect. The thresholds are: probably damaging (preservation time is greater than 450 my), possibly damaging (preservation time is between 200 my and 450 my), and probably benign (preservation time is less than 200 my).

c The MutPred2 prediction ranges from 0.0 and 1.0, in which a higher score indicates a greater propensity to be pathogenic.

d The MutationTaster score ranges from 0.0 to 215. It is derived from the Grantham Matrix for amino acid substitutions and shows the physicochemical difference between the original and the mutated amino acid.

e Fathmm‐XF predictions are given as *p*‐values in the range 0–1: values above 0.5 are predicted to be deleterious, while those below 0.5 are predicted to be neutral or benign.

### 3.3 Identification by QMPSF of a new *CLDN16* deletion

**FIGURE 2:**
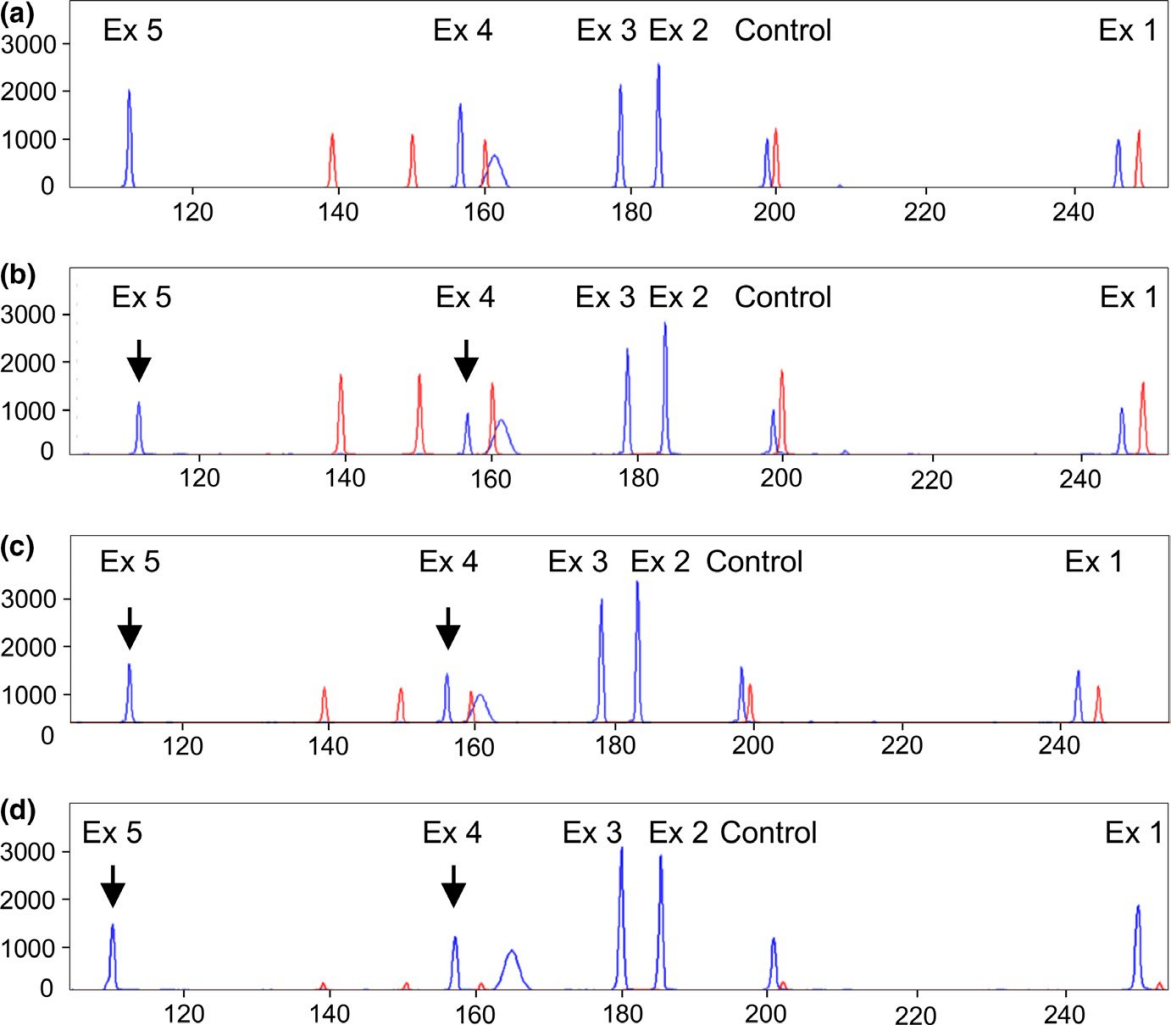
Detection of the *CLDN16* deletion using a QMPSF assay. Panels show the QMPSF electropherograms of: (a) normal control, (b) proband, (c) brother, and (d) father. Blue peaks and red peaks correspond to *CLDN16* exons 1–5 fragments and molecular weight markers, respectively. The Y‐axis indicates fluorescence in arbitrary units, and the X‐axis displays the size in base pairs. The heterozygous deletion is easily detected by a reduction of the peaks corresponding to exons 4 and 5 (arrows) in the proband, his brother and father compared to a normal control

Direct sequencing of *CLDN16* revealed only a mutated allele, therefore, since FHHNC is an autosomal recessive disease, we suspected that the mutation in the other allele was a large heterozygous deletion in this gene. In order to detect a potential heterozygous deletion of *CLDN16* that could explain the clinical phenotype and to map precisely the deletion, we designed a QMPSF assay, a method based on simultaneous amplification of multiple short sequences under quantitative conditions. The results of this assay revealed a novel large deletion in the *CLDN16* gene of the patient that, at least, lacked exons 4 and 5, c.(840+25_?)del. As illustrated by Figure 2, exonic deletions were easily detected by a 50%decrease of the corresponding peaks; the probes designed for exons 4 and 5 presented half the expected signal in the patient, brother and father. In contrast, exons 1, 2, and 3 were not deleted. The patient's unaffected brother and father were found to be carriers of this deletion (Figure 2). This novel *CLDN16* deletion was submitted to ClinVar and it was included with accession number SCV001335520.

### 3.4 Modeling of claudin‐16 protein structure

**FIGURE 3:**
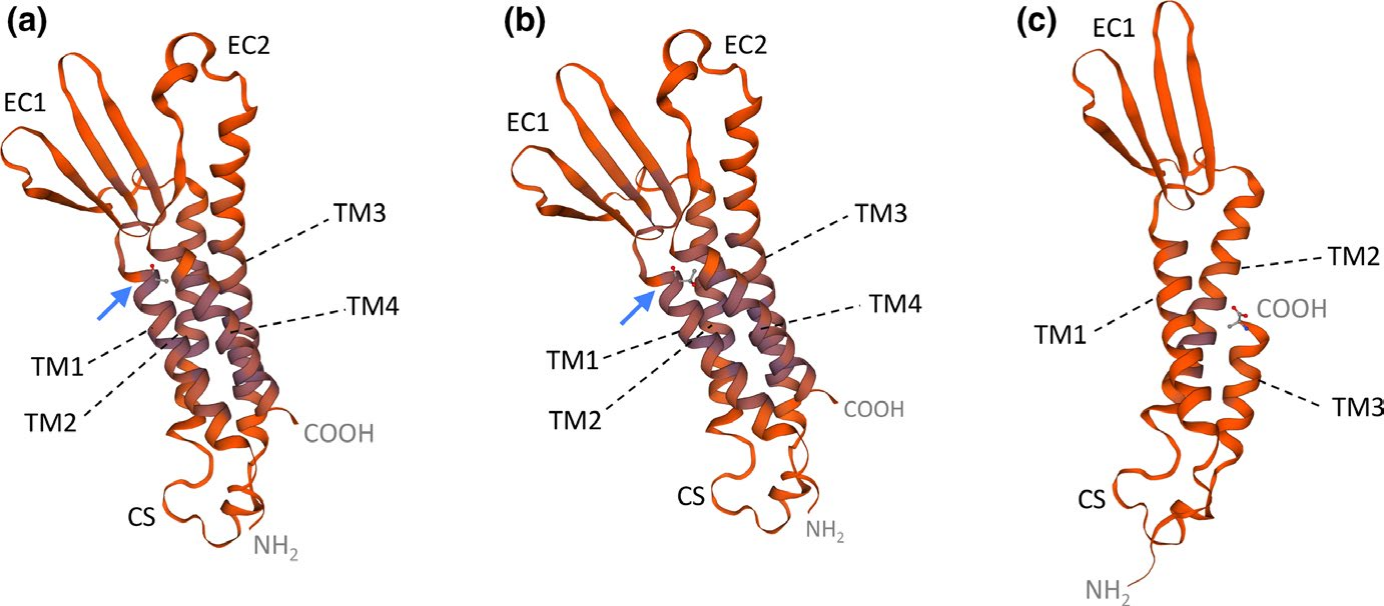
Predicted 3D structures of wild‐type and mutant claudin‐16 proteins generated using homology modeling with SWISS‐MODEL.TM1 to 4, transmembrane domains 1 to 4; EC1 and EC2, extracellular loops 1 and 2; CS, cytoplasmic segment. (a) Wild type claudin‐16. (b) Mutant p.(Ala93Thr). Blue arrows indicate the location of the original alanine 93 residue and missense mutation p.(Ala93Thr). The NH_2_ and COOH termini correspond to threonine 70 and cysteine 255, respectively. (c) Deletion mutant lacking part of TM3, EC2, TM4, and COOH terminus. The COOH terminus corresponds now to alanine 197. The models are based on template 5b2 g.2 of claudin‐14

To analyze the structural impact of the two *CLDN16* mutations detected in the patient, the 3D structure of the claudin‐16 protein was modeled based on the solved protein structure of claudin‐4 using SWISS‐MODEL. The results did not reveal any derangement in the structure of the mutant claudin‐16 protein with the missense mutation (Figure 3). The predicted 3D structure of the claudin‐16 protein encoded by the allele with the deletion of exons 4 and 5 resulted in the removal of nine amino acids of the third transmembrane domain, the second extracellular loop, the fourth transmembrane domain (encoded by exon 4) and the carboxy‐terminal cytoplasmic region containing a PDZ‐binding motif and potential phosphorylation sites (encoded by exon 5) (Figure 3).

## 4 DISCUSSION

FHHNC is a rare hereditary disease characterized by excessive renal losses of magnesium and calcium, bilateral nephrocalcinosis, and progressive chronic renal failure (Claverie‐Martin, 2015; Praga et al., 1995). Clinical observations and clearance studies in patients suggested that the primary defect was associated with reduced paracellular reabsorption of magnesium and calcium in the thick ascending loop of Henle (Rodriguez‐Soriano, Vallo, & Garcia‐Fuentes, 1987). The disease is caused by loss‐of‐function mutations of*CLDN16* (previously known as *PCLN1*) (type 1), (Simon et al., 1999) or *CLDN19* (type 2).The majority of Spanish patients with FHHNC are homozygous for *CLDN19* founder mutation p.(Gly20Asp), and they also have severe ocular defects (Konrad et al., 2006, Claverie‐Martin et al., 2013; Martin‐Nuñez et al., 2015). We examined the phenotype and genotype of a Spanish child with the typical characteristics of FHHNC and no ocular abnormalities. He suffered from polyuria and hyposthenuria. Bilateral nephrocalcinosis, mild hypomagnesemia, hypercalciuria, elevated fractional excretion of magnesium, increased parathyroid hormone, and decreased glomerular filtration rate were found. Genetic analysis of the proband and family members revealed two novel mutations of *CLDN16* in a compound heterozygous state, which confirmed the clinical diagnosis of FHHNC type 1.

The *CLDN16* gene is located on chromosome 3q27, and to date, 69 pathogenic mutations have been identified: 43 missense, 10 nonsense, 5 splicing, 5 small deletions, 2 small insertion, 2 small indels, 1 complex, and 1 gross deletion (Prot‐Bertoye & Houillier, 2020). Because of the autosomal recessive pattern of inheritance in FHHNC type 1, patients are homozygous or compound heterozygous for the particular mutations. Functional studies have shown that *CLDN16* mutations may cause partial or complete loss of claudin‐16 function (Hou et al., 2005; Kausalya et al., 2006; Konrad et al., 2008; Müller et al., 2003). The majority of mutant claudin‐16 proteins display normal trafficking to the cell membrane, but others remain in the endoplasmic reticulum, Golgi apparatus, or lysosomes. Even mutant proteins that are correctly localized to the tight junction frequently show defective magnesium paracellular transport (Hou et al., 2005; Konrad et al., 2008). Hou and colleagues have shown that claudin‐16 interacts with claudin‐19 conferring cation selectivity to the tight junction in a synergistic manner (Hou et al., 2008). Mutations that disturb this interaction cause loss of cation selectivity.

Direct sequencing of *CLDN16* exons identified heterozygous missense variant c.277G>A; p.(Ala93Thr) in our patient, which was inherited from his mother. This very rare variant, which results in the replacement of an alanine residue for threonine, has not been described previously in FHHNC. The substitution of a hydrophobic amino acid for a polar amino acid in the first transmembrane domain of claudin‐16 could lead to misfolding or defects in membrane insertion. Therefore, p.(Ala93Thr) could affect the formation of claudin‐16/claudin‐19 tight junctions. We consider this variant is a pathogenic mutation based on the following: (1) it affects a highly conserved amino acid residue, (2) bioinformatics analysis predicts deleterious consequences, (3) it's extremely low frequency, and (4) it results in a decrease of protein stability. However, this would have to be confirmed experimentally by functional analysis. Most *CLDN16* mutations affect the two extracellular loops of the protein, and only two previously reported missense mutations are located in the first transmembrane domain, p.(Cys80Tyr) and p.(Gly88Glu) (Prot‐Bertoye & Houillier, 2020). Functional analysis of p.(Gly88Glu) has revealed that this mutation results in a complete loss of function (Konrad et al., 2008).

Since only one heterozygous mutation of *CLDN16* could not explain the FHHNC phenotype of our patient, we used a QMPSF assay designed by us to detect the mutation in the other allele. The results showed a heterozygous deletion of exons 4 and 5 that was inherited from his father. The claudin‐16 protein encoded by this mutant would lack part of the third transmembrane domain, the second extracellular loop, the fourth transmembrane domain, and the cytoplasmic carboxy terminus, which plays an important role in protein stability and trafficking to the tight junction (Itoh et al., 1999; Müller et al., 2003). Therefore, we assumed that this deletion induces a complete loss of function of the mutant claudin‐16 protein. Only one large homozygous deletion in the *CLDN16* gene of a patient with FHHNC has been reported in the literature (Yamaguti et al., 2015). This deletion was detected using a multiplex ligation‐dependent probe amplification assay, and included exons 2 to 5. *CLDN16* large deletions may be underestimated, and we suggest that in cases where only one mutated allele is detected, the genetic analysis should include a method to detect large deletions.

The pathogenesis of chronic renal disease in patients with FHHNC remains unclear. The hypercalciuria and nephrocalcinosis present in these patients may contribute to progression to end‐stage renal disease, however, a suitable correlation has not been found (Praga et al., 1995). Moreover, not all hereditary tubulopathies characterized by nephrocalcinosis lead to end‐stage renal disease. Other factors related to the progression of chronic kidney disease could be the activation of the inflammasome by crystal nephropathy or the anomalies caused by the defective claudin‐16 function early in the development of tubular tight junctions (Godron et al., 2012; Claverie‐Martin et al., 2015). Konrad and colleagues have suggested a genotype‐phenotype correlation related to the progression of renal failure in FHHNC patients with *CLDN16* mutations (Konrad et al., 2008). They found that the progression of renal failure is significantly faster in patients with complete loss‐of‐function mutations in both alleles as compared with patients with partial loss‐of‐function mutations in one or both alleles. As most patients with FHHNC, our patient presented the typical characteristics of FHHNC, including moderate chronic kidney disease early in infanthood. However, during the last 7 years of follow‐up, he showed a slow deterioration in renal function (eGFR from 56–40 ml/min/1.73 m^2^) in comparison with other patients (Weber et al., 2001). This suggests that at least one of the two mutations detected in the patient has a partial loss of function. Since the large deletion obviously has a complete loss of function, we speculate that p.(Ala93Thr) has a residual claudin‐16 function that delays progression of renal failure.

In conclusion, we report the clinical and genetic features of a Spanish boy with early onset of FHHNC symptoms but slow deterioration in renal function. By applying direct DNA sequencing and a QMPSF analysis designed by us, two novel compound heterozygous pathogenic variants of *CLDN16* were identified; a missense mutation,c.277G>A; p.(Ala93Thr), in one allele and a large deletion, c.(840+25_?)del, in the other allele. The missense mutation affects the first transmembrane domain of claudin‐16 and probably conserves a residual function, while the deletion involves exons 4 and 5 and most likely leads to a complete loss of function. These mutations were inherited from his mother and father, respectively. Our study provides further insights into the molecular basis of FHHNC. Moreover, the QMPSF assay described here should facilitate the genetic diagnosis of FHHNC.

## CONFLICT OF INTEREST

The authors declare that they have no competing interests.

## AUTHOR CONTRIBUTION

AG‐C designed and implemented the QMPSF assay. AP‐R and M‐VP performed PCR amplifications and analyzed the DNA sequences. AP‐R, ER‐T, and FC‐M contributed to the bioinformatics analysis. GA and LM collected the patient's data and followed up the case. FC‐M, AG‐C, and GA wrote the paper. FC‐M and GA acquired funding. All authors read and approved the final version of the manuscript.
